# The dietary approaches to stop hypertension (DASH) dietary pattern in childhood in relation to cardiometabolic risk in adolescence and early adulthood in the ALSPAC birth cohort

**DOI:** 10.1017/S136898002400048X

**Published:** 2024-03-21

**Authors:** Panayiotis Loizou, Caroline M Taylor, Genevieve Buckland

**Affiliations:** 1 Bristol Medical School, University of Bristol, Bristol, UK; 2 Centre for Academic Child Health, Canynge Hall, 39 Whatley Road, Bristol Medical School, University of Bristol, Bristol BS8 2PS, UK

**Keywords:** Dietary approaches to stop hypertension dietary pattern, Avon Longitudinal Study of Parents and Children, Children and adolescents, Cardiometabolic risk score, Dietary approaches to stop hypertension diet score, Prospective cohort study

## Abstract

**Objective:**

To investigate the relationship between the dietary approaches to stop hypertension (DASH)-style dietary patterns in childhood and cardiometabolic risk (CMR) in adolescence/early adulthood.

**Design:**

Data were obtained from the Avon Longitudinal Study of Parents and Children (ALSPAC) prospective cohort. Diet diary data collected at 7, 10 and 13 years were used to calculate DASH-style diet scores (DDS). Multivariable linear regression models were used to investigate the associations between the DDS at 7, 10 and 13 years and CMR scores, calculated at 17 and 24 years.

**Setting:**

The ALSPAC cohort included children born in south-west England in 1991–1992.

**Participants:**

Children with complete dietary, covariate and cardiometabolic data at 17 (*n* 1,526) and 24 years (*n* 1,524).

**Results:**

A higher DDS at 7 and 10 years was negatively associated with CMR scores at 17 years (*β* = –0·64 (95 % CI –1·27, –0·006), *P*_trend_=0·027 for fifth *v*. first DDS quintile at 7 years; *β* = –0·73 (95 % CI –1·35, –0·12) and *P*_trend_=0·037 for fifth *v*. first DDS quintile at 10 years) and at 24 years (*β* = –0·92 (95 % CI –1·49, –0·34) *P*_trend_ = 0·001 for fifth *v*. first DDS quintile at 7 years; *β* = –0·60 (95 % CI –1·20, –0·05) *P*_trend_ = 0·092 for fifth *v*. first DDS quintile at 10 years). No associations were found between the DDS at 13 years and CMR score at 17 and 24 years.

**Conclusion:**

Greater adherence with a DASH-style diet during childhood was associated with better cardiometabolic health in adolescence/adulthood in the ALSPAC cohort. The components of the DASH diet could be recommended to improve children’s cardiometabolic health.

Cardiometabolic diseases (CMD) are the leading cause of mortality globally^(1)^. These include CVD, diabetes mellitus and chronic kidney conditions^(2)^. The pathogenesis of CMD can begin during childhood or adolescence^(3)^ due to the early onset of various cardiometabolic risk (CMR) factors including elevated blood pressure (BP), dyslipidaemia, obesity, endothelium dysfunction and abnormal glucose control^(4)^. Adopting healthier lifestyle practices, including healthy dietary habits, has been shown to reduce the likelihood of CMR factors developing and persisting into adulthood^(5–9)^. However, there is still limited robust evidence on how different diet patterns in children impact overall cardiometabolic health^(10,11)^. This area of research is important because childhood is a period when dietary habits are established and become embedded and can then influence future health.

Analyses of dietary patterns are useful to understand the complex relationship between the combination of different foods and nutrients and their interactions and cardiometabolic health^(12)^. Previous research has demonstrated that unhealthy or western dietary patterns (high in processed foods, red meat, refined grains, salt and sugar) are positively associated with different CMR factors (adiposity and raised TAG, insulin and glucose levels)^(10)^. Conversely, healthy dietary patterns high in plant-based foods, healthy oils and seafood have been linked to better cardiometabolic health^(10)^.

The Dietary Approaches to Stop Hypertension (DASH) diet was developed in the 1990s from research trials aiming to treat hypertension without medication^(13,14)^. These randomised control feeding trials showed that the DASH diet significantly reduced both systolic blood pressure (SBP) and diastolic blood pressure (DBP) in hypertensive and normotensive individuals^(13,14)^. The DASH diet has the key properties of a cardioprotective diet, advocating a high consumption of fruit, vegetables, legumes, whole grains, low-fat dairy products and nuts and low consumption of sugars, sodium and red and processed meats^(15)^. Subsequent experimental and observational studies have shown that the DASH diet can reduce the risk of other cardiovascular risk factors in addition to hypertension, such as excess adiposity, insulin resistance and high levels of cholesterol^(16)^. Greater adherence to the DASH diet in adults is also linked to reduced risk of developing cardiometabolic diseases and mortality from different chronic conditions^(16,17)^, and consequently this healthy dietary pattern is now recommended by a number of professional associations such as the American Heart Association^(18)^ and the American Diabetes Association^(19)^.

To aid epidemiological research into the DASH diet, the key features of this diet have been operationalised into DASH diet scores (DDS) using different methodological approaches^(20,21)^. DASH scores represent the degree of adherence to this dietary pattern and have been used extensively in adults to study their association with cardiometabolic diseases and their risk factors^(16,22–24)^. However, epidemiological studies examining how the DASH diet effects individual CMR factors in children and adolescents are limited and have produced inconsistent findings^(25)^. A cross-sectional study based on data from the UK 2008–2016 National Diet and Nutrition Survey did not observe any associations between the DASH diet and waist circumference, BP, glucose, HDL-cholesterol or TAG levels in the 11–30-year-olds^(15)^. In contrast, two cohort studies found negative associations between high adherence to the DASH diet and BP and BMI in adolescents^(26,27)^. A recent cohort study in Mexico among children and young adults reported that a higher DASH score was associated with a decrease in insulin resistance, but not other cardiometabolic markers^(28)^, although combined cardiometabolic risk was not assessed.

The above-mentioned research using National Diet and Nutrition Survey data did study the effect of the DASH diet on a composite metabolic risk *z*-score (generated from five metabolic risk factors), and although they found an association in the middle-aged adults, there was no evidence for an association in adolescents or young adults^(15)^. CMR scores provide an indication of overall cardiometabolic risk by combining measurements from multiple cardiometabolic markers such as lipids, measures of adiposity, BP and metabolism, although to date there is no single official definition of a CMR score^(29)^. CMR scores are particularly useful when studying cardiometabolic health in children as they accumulate potentially small variations in a range of risk factors that may be too subtle to indicate risk on their own in younger age groups^(29,30)^. Our group previously found that a Mediterranean-style diet in childhood was related to a better cardiometabolic profile (lower CMR score) in early adulthood^(31)^.

However, there is still very limited research from prospective cohort studies in children investigating the effects of the DASH diet on cardiometabolic health using composite CMR scores. Therefore, this study investigated the relationship between the DASH-style dietary pattern in children and overall CMR in adolescents and young adults in the ALSPAC cohort.

## Methods

### Study population

**Fig. 1:**
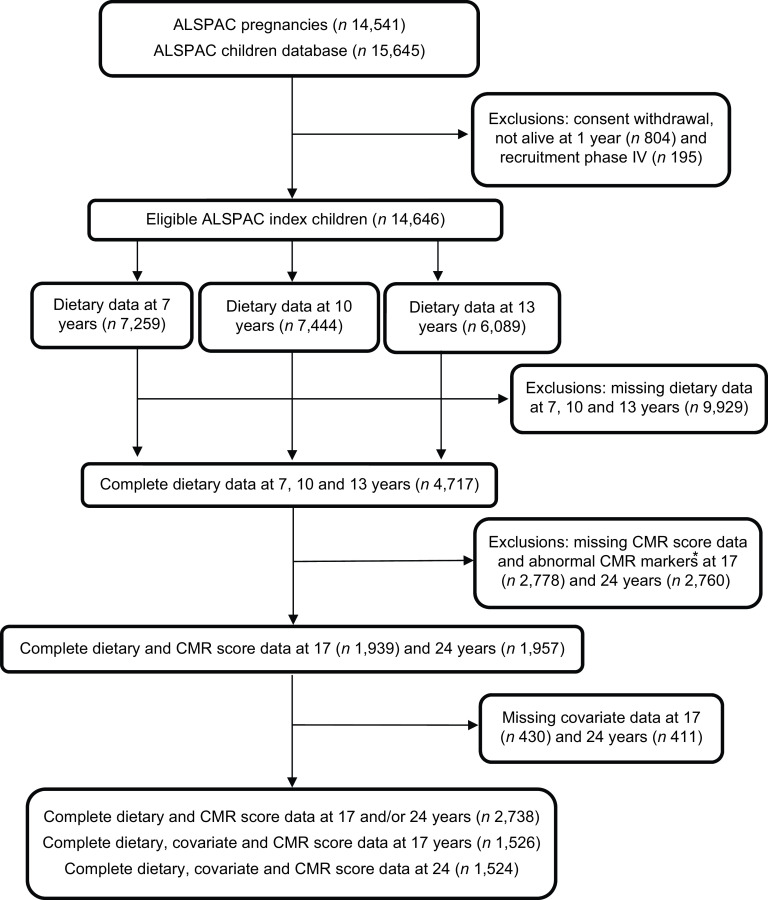
Study flow diagram for the population from the ASLPAC cohort. *Excluded participants diagnosed with diabetes (*n* 17 at 17 years; *n* 17 at 24 years), being on insulin treatment (*n* 15 at 17 years; *n* 15 at 24 years), having a fasting glucose level of ≥7 mmol/L (*n* 14 at 17 years; *n* 44 at 24 years) and extreme outliers (>4SD from the mean), (*n* 56 at 17 years; *n* 46 at 24 years. ALSPAC, Avon Longitudinal Study of Parents and Children.

The current study uses data from the on-going ALSPAC prospective birth cohort^(32–34)^. The ALSPAC website (www.alspac.bris.ac.uk) provides full study methodological details. In brief, 14 541 pregnant women from the south-west of England were initially recruited from 1991 to 1992^(32,33)^. From these pregnant women, 13 988 children were alive at 1 year of age. Additional children were recruited into the cohort during the 7 year assessment clinic and during opportunistic contacts thereafter, which resulted in a total of 14 901 children^(34)^. The sample size of the current study was 14 646 children after excluding withdrawals of consent during follow-up and triplet and quadruplet pregnancies for confidentiality reasons (Fig. 1). Data were collected from the parents and children during regular follow-ups using questionnaires, assessment clinics and medical records. Data are stored and managed using Research Electronic Data Capture (REDCap) electronic data capture tools hosted at the University of Bristol^(35)^. REDCap is a secure, web-based software platform designed to support data capture for research studies. The study website contains details of all the data that is available through a fully searchable data dictionary and variable search tool (http://www.bristol.ac.uk/alspac/researchers/our-data/).

### Dietary assessment

Children were asked to attend the study’s assessment clinics when they were 7, 10 and 13 years old (mean age 7·5 years (sd 0·31), 10·6 years (sd 0·22) and 13·8 years (sd 0·19) years, respectively). Prior to the clinics, parents/caregivers were sent 3-day diet diary record (3-DDR) sheets to record the children’s dietary intake over 3 days (one weekend day and two weekdays of their choice). 3-DDR was completed by the parents when the children were 7 years old and by the children at 10 and 13 years old along with parental assistance. Standard household measures (bowls, cups, teaspoons, packet sizes, dessert spoons, etc.) in grams were used to record portion sizes^(36)^. Information regarding the details and the amount of food and drinks consumed was recorded along with a description of any leftovers. When the children and parents attended the assessment clinic, a nutritionist checked the 3-DDR for completeness and discrepancies and also clarified portion sizes. Further details on the dietary assessment methods in ALSPAC have been previously published^(36)^. The data from the 3-DDR were coded and linked to food composition tables using Diet In Data Out. Household measurements and portion sizes were used to convert food and drinks into weighted dietary intakes. McCance and Widdowson’s British food composition data were used to calculate nutrient intakes^(37)^.

The plausibility of reported total dietary intake was calculated individually based on the ratio of energy intake (EI) to estimated energy requirements and their 95 % CI^(38)^. Dietary data obtained from the 3-DDRs were available for 7,259 7-year-olds, 7,444 10-year-olds and 6,089 13-year-olds, with complete dietary data at all three ages available for 4,717 children (see Fig. 1).

### DASH diet score

The DASH dietary score outlined by Jones et al. in 2008^(20)^ was used to assess how closely the children’s diets at 7, 10 and 13 years aligned to a DASH-style dietary pattern (Table 1). This criteria for assessing adherence to the DASH dietary pattern were used, in place of other published methods, because it has been previously associated with incident stroke and CVD in a British population^(20)^. This method takes into account eight dietary components including five healthy components (fruit, vegetables, nuts and legumes, wholegrains and low-fat dairy) and three unhealthy components (red and processed meat, sodium and non-milk extrinsic sugars). Additional information regarding foods included within each of the DASH dietary components can be found in the online supplementary material, Supplementary Table 1. The energy density method was applied to calculate each participant’s intake of the separate DASH foods/nutrients relative to their total EI (grams per day of food or nutrient/total EI in kJ per day) × (1000)^(39)^. Subsequently, scores of 1–5 were assigned according to quintiles of intakes of each separate food group/nutrient. The five healthy components of the DASH diet were scored from 1 to 5, with the greatest consumers having a score of 5. The three unhealthy components of the DASH diet were scored inversely, with the lowest consumers (first quintile) having a score of 5. The resulting quintiles scores for all eight dietary components were summed to create an overall Dash Diet Score (DDS) for each child at all ages, potentially ranging from 8 to 40. The DDS was divided again into five quintiles, with quintiles 1 and 5 represented low and high accordance to the DASH diet, respectively.

**Table 1:** Scoring criteria for the DASH-diet score (DDS) based on Jones et al.,^(20)^ using the energy density method for the quintiles 1–5

Dietary components	Scoring criteria				
	Q1	Q2	Q3	Q4	Q5
	Points assigned				
**Food groups where greater consumption received higher score**					
Fruit	1	2	3	4	5
Vegetables	1	2	3	4	5
Nuts and legumes	1	2	3	4	5
Wholegrains	1	2	3	4	5
Low-fat dairy	1	2	3	4	5
**Food groups where lowest consumption received higher score**					
Red and processed meat	5	4	3	2	1
NMES	5	4	3	2	1
Na	5	4	3	2	1

DASH, dietary approaches to stop hypertension; NMES, non-milk extrinsic sugar; Q, quintile.

### Cardiometabolic marker assessments

Cardiometabolic markers, including BP, blood biomarkers and anthropometric measurements, were measured during assessment clinics that the participants attended at 17 years (mean 17·8 (s d 0·3) years) and at 24 years (mean 24·5 (s d 0·8) years). Seated BP was measured using BP monitors (Omron 705-IT and Omron IntelliSense-M6). Appropriate cuff sizes were used for each participant to measure SBP and DBP twice on the right arm and the mean value was calculated. Mean arterial blood pressure was calculated as 1/3(SBP) + 2/3(DBP)^(40)^. Participants were required to fast overnight or for 6–8 h before blood samples were taken. Plasma lipids, including HDL-cholesterol, LDL-cholesterol and TAG, were examined following the standard Lipid Research Clinics Protocol^(41)^. The homoeostatic model assessment of insulin resistance (HOMA-IR) was determined by using derived glucose and insulin measurements ((glucose (mg/dl) × insulin (mU/l))/405)^(42)^. Height and weight were measured using a stadiometer (Holtain Ltd) and a body composition scale (Tanita) to the nearest 0·1 cm and 0·1 kg, respectively. Fat mass was measured using a dual-energy X-ray absorptiometry digital scanner (GE Medical Systems). BMI (kg/m^2^) and fat mass index (FMI) (kg/m²) were calculated based on these anthropometric measurements.

### Cardiometabolic risk (CMR) score

A composite CMR score was calculated based on previously published methods used to study cardiometabolic risk in ALSPAC adolescents and young adults^(30)^. This CMR score incorporates the most commonly included components used in research using continuous CMR scores measured in childhood, according to a scoping review^(29)^. In addition, this CMR score has previously been used to measure associations with several major dietary patterns within the same cohort^(30,31)^. Before calculating the CMR score, participants were excluded if they were diagnosed with diabetes (*n* 17 at 17 years; *n* 17 at 24 years), were on insulin treatment (*n* 15 at 17 years; *n* 15 at 24 years) and/or had a fasting glucose level of ≥7 mmol/L (*n* 14 at 17 years; *n* 44 at 24 years) due to potential problems of using HOMA-IR to assess insulin sensitivity in participants with diabetes^(43)^. In addition, participants were excluded from the analysis if they had extreme/implausible values on any of the CMR score components, which was determined by examining histograms and using exclusion criteria of >4SD from the mean (*n* 56 at 17 years; *n* 46 at 24 years). The CMR score combines each participant’s measured values for six cardiometabolic markers (FMI, HDL-cholesterol, LDL-cholesterol, TAG, mean arterial blood pressure and HOMA-IR).

Each CMR marker was first converted into sex-specific *z*-scores in order to standardise their units (individual’s marker value minus sex-specific sample mean)/sex-specific sample (s d). HDL-cholesterol was multiplied by –1 to align the direction of values for increased risk with the other components. The sum of all the *z*-scores from the six cardiometabolic markers was calculated to create an overall CMR score for each child at 17 and at 24 years. Higher CMR scores represented increased overall CMR relative to the lower CMR scores. A total of 1,939 and 1,957 participants at 17 and 24 years had complete dietary and CMR score data after exclusions.

### Covariates

Data on covariates were collected by the ALSPAC team using medical reports, questionnaires completed during pregnancy and follow-up and at periodic assessment clinics. Maternal age at delivery was calculated using the maternal date of birth and the child’s date of birth. Family highest social class was determined by combining maternal and paternal social class which was based on the 1991 Office of Population Censuses and Surveys occupation-based classification system using the current or last job at 32 weeks of gestation. Moderate-to-vigorous physical activity at 11 years was collected using an Actigraph AM7164 2·2 accelerometer (Actigraph, LLC), which was placed around the participant’s waist for 1 week. The accelerometer was then used to calculate the mean minutes per day when participants were vigorously physically active (>3600 accelerometer counts per minute).

Covariates were initially considered as potential confounders of the DASH diet and CMR association based on previous research^(15,25–27)^ and a priori knowledge of diet-cardiometabolic health pathways and then through the use of statistical tests (Pearson correlation coefficient test for continuous covariates and the Kruskal–Wallis test for categorical covariates) to assess the association between covariates and the DDS and the CMR score. A significance cut-off value of approximately <0·1 for the above tests was used to guide the selection of appropriate covariates to include in the final multivariable regression models. Based on these criteria, seven covariates were identified including mothers’ age at delivery (categorical: ≤16–24, 25–29, 30–34 and ≥35 years), mothers’ highest education level (categorical: certificate of secondary education (CSE)/vocational training, O-level and A-level/degree), family highest social class (categorical: I/II and III/IV/V), plausibility of energy reporting at age 7, 10 and 13 years (categorical: under-reporting, plausible reporting and over-reporting) and moderate-to-vigorous physical activity at 11 years (categorical: <20 and ≥20 mins).

### Statistical analysis

STATA version 17·0 (Stata Corporation) was used to perform all statistical analyses. Proportions were used to describe categorical variables and mean (s d) or median (IQR) were used for parametric and non-parametric continuous variables, respectively. Kruskal-Wallis and Pearson correlation coefficient tests were used to assess differences between categorical and continuous variables, respectively. The baseline characteristics of the ALSPAC children were compared between participants included (*n* 2,738) and excluded (*n* 11 908) in the present study (participants included had complete dietary data from 7, 10 and 13 years and outcome data at 17 and/or 24 years). The medians (IQR) of cardiometabolic markers at 17 and 24 years were also compared between participants included and excluded in the present analysis.

Baseline characteristics of the participants (*n* 2,738) were described according to the DDS quintiles at 7, 10 and 13 years. The data on the six individual CMR score components at 17 years (*n* 1,939) and 24 years (*n* 1,957) were also described according to DDS quintiles. The correlation for the continuous DDS between 7, 10 and 13 years was measured using partial Pearson correlation coefficients adjusted for dietary misreporting.

Adjusted and unadjusted multivariable linear regression models were used to examine the relationships between the categorical DDS at 7, 10 and 13 years and continuous CMR score at 17 (*n* 1,526) and 24 (*n* 1,524) years, in a complete case analysis. The CMR score was assessed as a continuous variable (per 1-unit increase). The standardised regression beta-coefficients (*ß*) and 95 % CI represent the estimated mean change in CMR *z*-score associated with each DDS quintile (first quintile as reference category). An unadjusted model, a partially adjusted model (model 1) and a fully adjusted model (model 2) were generated to allow for potential confounders. The partially adjusted model was adjusted for sex and plausibility of energy reporting at the age that dietary data was collected. The fully adjusted model was further adjusted for grouped age of the mother at delivery, mothers’ highest education attainment, family’s highest social class and moderate-to-vigorous physical activity. The variance inflation factor was used to test for multicollinearity. Multivariable linear regression models were also used to assess the association between the DDS (per five-point increment) and the individual CMR score components (FMI, HDL-cholesterol, LDL-cholesterol, TAG, mean arterial blood pressure and HOMA-IR) and additional CMR factors (BMI, waist circumference, SPB, DBP, total cholesterol, insulin and glucose). The results are presented for both sexes together because there was no evidence that sex modified the association between the DDS and CMR score (*P* values >0·10 from the test comparing models with and without an interaction term between the DDS and sex). In sensitivity analyses, the main analysis of the association between the DDS at all three ages and CMR score at 17 and 24 was restricted to plausible dietary reporters.

## Results

The final sample size was 1,939 (50 % female) participants with DDS and CMR score data at 17 years and 1,957 (57 % female) participants with DDS and CMR score data at 24 years (Fig. 1). In general, participants included in the study were more likely to be female, have a higher EI, maternal educational attainment and household social class and better cardiometabolic profile at 17 and 24 years than the participants excluded from the study (see online supplementary material, Supplementary Tables S2 and S3).

The mean DDS at 7 years was 23·5 (sd 4·7) and ranged from 9 to 38 points. The mean DDS at 10 years was 23·4 (sd 4·7) and ranged from 10 to 38 points. The mean DDS at 13 years was 23·3 (sd 4·7) and ranged from 9 to 36 points. The correlation coefficient for the continuous DDS between 7 and 10 years was 0·45 (95 % CI: 0·43, 0·47), between 7 and 13 years it was 0·39 (95 % CI: 0·36, 0·41) and between 10 and 13 years it was 0·42 (95 % CI 0·40, 0·45), indicating moderate correlation of this dietary pattern throughout this period. The CMR *z*-score at 17 ranged from –7·72 to 16·77 and at 24 years ranged from –9·38 to 17·84. Table 2 represents the baseline characteristics of the 2,738 participants with complete dietary and CMR score data at 17 and/or 24 years according to the DDS quintiles at 7, 10 and 13 years. Participants in the fifth compared with the first DDS quintile at most ages were more likely to be female, had lower EI, better household social class, lower rates of dietary misreporting, higher maternal education level and lower maternal BMI. Table 3 shows the cardiometabolic characteristics for the participants at 17 (*n* 1,939) and 24 (*n* 1,957) years with complete dietary and CMR score data. FMI and HOMA-IR z-scores at 17 years were lower in participants with higher DDS scores at 7 and 10 years. Participants in the top DDS quintile at 7, 10 and 13 years also had lower FMI, mean arterial blood pressure and HOMA-IR scores and higher HDL-cholesterol scores at 24 years.

**Table 2:** Characteristics of the ALSPAC population with complete dietary and cardiometabolic risk (CMR) score data at 17 and/or 24 years, based on quintile 1 and 5 of the DASH-diet score (DDS) in children at 7, 10 and 13 years (*n* 2,738)

Characteristics of the ALSPAC population	Total n	DDS at 7 years					DDS at 10 years					DDS at 13 years				
		Quintile 1		Quintile 5		*P*-value*	Quintile 1		Quintile 5		*P*-value*	Quintile 1		Quintile 5		*P*-value*
		Mean	sd	Mean	sd		Mean	sd	Mean	sd		Mean	sd	Mean	sd	
Age (years)	2,738	7·5	0·28	7·5	0·31	0·893	10·6	0·24	10·6	0·20	0·028	13·8	0·18	13·8	0·18	0·014
		Median	IQR	Median	IQR		Median	IQR	Median	IQR		Median	IQR	Median	IQR	
Children BMI (kg/m^2^)	2,719	15·7	14·85–17·09	15·7	14·79–16·84	0·620	17·5	15·88–19·66	17·2	15·91–19·21	0·574	19·7	17·95–21·95	19·5	17·94–21·56	0·507
Energy intake (kJ/d)	2,738	7,061	6,247–7,929	7,212	6,407–8,065	0·026	7,655	6,639–8,909	7,631	6,824–8,613	0·043	8,114	6,776–9,733	7,885	6,751–9,152	0·264
Mothers’ BMI (kg/m^2^)	2,449	22·4	20·7–24·7	21·5	20·3–23·5	<0·001	22·4	20·5–24·7	21·5	20·3–23·2	0·001	22·4	20·6–24·8	21·5	20·1–23·4	<0·001
		%		%			%		%			%		%		
Sex (male)	2,738	13·0		7·3		0·393	10·8		8·8		0·129	11·0		7·1		<0·001
Mothers age at delivery (>30 years)	2,631	11·3		9·3		<0·001	10·0		11·6		<0·001	9·6		11·9		<0·001
Mothers highest education (A-level/degree)†	2,594	9·8		10·3		<0·001	7·9		13·2		<0·001	8·1		12·9		<0·001
Highest household social class (grade I/II)‡	2,531	6·0		7·4		<0·001	5·5		9·6		<0·001	4·7		9·1		<0·001
MVPA at 11 years (≥20 min/d)	2,334	12·9		7·7		0·243	9·8		10·4		0·278	10·6		10·7		0·074
Accuracy of energy reporting (misreporting)§	2,730	6·8		3·0		0·005	9·9		4·6		<0·001	17·2		9·0		0·725

ALSPAC, Avon Longitudinal Study of Parents and Children; DASH, dietary approaches to stop hypertension; IQR, interquartile range; MVPA, moderate-vigorous physical activity.

* *P* values were calculated using the Kruskal–Wallis test.

†‡§ Categorical variables including mothers highest education (A-level or degree), highest household social class (grade I and II) and accuracy of energy reporting (misreporting) were recategorised for the purpose of the above table.

**Table 3:** Cardiometabolic risk factors of the ALSPAC participants with complete dietary and cardiometabolic risk (CMR) score data at 17 and 24 years (*n* 1,939 at 17 years; *n* 1,957 at 24 years), based on quintile 1 and 5 of the DASH-diet score (DDS) in children at 7, 10 and 13 years

Cardiometabolic risk factors of the ALSPAC participants	Total n	DDS at 7 years					DDS at 10 years					DDS at 13 years				
		Quintile 1		Quintile 5		*P* value*	Quintile 1		Quintile 5		*P* value*	Quintile 1		Quintile 5		*P* value*
		Median	IQR	Median	IQR		Median	IQR	Median	IQR		Median	IQR	Median	IQR	
**CMR factors at 17 years**																
FMI (kg/m^2^)	1939	5·9	3·36–8·58	4·8	2·79–6·93	0·004	5·7	3·18–8·19	4·9	2·84–6·79	0·055	5·3	2·91–8·05	5·5	3·59–7·33	0·519
HDL-cholesterol (mmol/l)	1939	1·2	1·04–1·44	1·3	1·07–1·46	0·106	1·2	1·05–1·44	1·3	1·09–1·51	0·120	1·2	1·04–1·44	1·3	1·10–1·48	0·274
LDL-cholesterol (mmol/L)	1939	2·1	1·70–2·49	2·0	1·66–2·57	0·434	2·1	1·67–2·49	2·0	1·65–2·46	0·755	2·0	1·65–2·47	2·1	1·65–2·45	0·725
TAG (mmol/l)	1939	0·7	0·61–0·98	0·8	0·59–0·96	0·905	0·7	0·59–0·92	0·7	0·59–0·91	0·317	0·7	0·61–0·94	0·8	0·60–0·93	0·919
MAP (mmHg)	1939	81·3	77·50–87·00	81·5	76·50–86·17	0·747	81·5	77·50–86·17	80·6	76·17–85·25	0·326	82·0	78·17–86·50	80·8	77·00–84·67	0·064
HOMA-IR	1939	1·5	1·13–2·16	1·4	1·00–1·93	0·007	1·6	1·12–2·14	1·4	1·00–1·84	0·005	1·5	1·06–2·13	1·4	1·03–1·87	0·268
**CMR factors at 24 years**																
FMI (kg/m^2^)	1957	7·4	5·46–9·96	6·4	4·87–8·43	<0·001	6·7	5·24–9·04	6·2	4·84–7·95	<0·001	7·1	5·35–9·38	6·5	5·09–8·52	0·176
HDL-cholesterol (mmol/l)	1957	1·5	1·23–1·75	1·6	1·33–1·85	0·003	1·5	1·25–1·77	1·6	1·31–1·87	0·009	1·5	1·23–1·75	1·6	1·32–1·85	0·003
LDL-cholesterol (mmol/l)	1957	2·4	1·94–2·89	2·3	1·83–2·83	0·233	2·4	2·04–2·88	2·3	1·91–2·75	0·139	2·4	1·94–2·89	2·3	1·86–2·86	0·325
TAG (mmol/l)	1957	0·8	0·65–1·14	0·8	0·66–1·07	0·627	0·8	0·64–1·12	0·8	0·66–1·06	0·560	0·8	0·65–1·13	0·8	0·64–1·08	0·274
MAP (mmHg)^†^	1957	83·0	78·56–88·67	81·1	76·22–87·67	0·035	82·7	77·94–88·39	81·5	76·39–87·28	0·038	83·3	78·44–88·56	81·1	76·44–86·22	<0·001
HOMA-IR^‡^	1957	1·8	1·23–2·59	1·6	1·15–2·32	0·003	1·7	1·19–2·50	1·5	1·07–2·20	<0·001	1·8	1·23–2·56	1·6	1·15–2·19	0·059

ALSPAC, Avon Longitudinal Study of Parents and Children; DASH, dietary approaches to stop hypertension; IQR, interquartile range; CMR, cardiometabolic risk; IQR, interquartile range (25th and 75th percentile); FMI, fat mass index; MAP, mean arterial blood pressure; HOMA-IR, homeostatic model assessment of insulin resistance.

* *P* values were calculated using the Kruskal–Wallis test.

† MAP = (1/3(SBP)) + (2/3(DBP)).

‡ HOMA-IR = (fasting plasma glucose (mg/dl)) × (fasting plasma insulin (mU/L))/405).

Tables 4 and 5 show beta coefficients, 95 % CI and *P* values for the unadjusted, partially adjusted and fully adjusted associations between the DDS in quintiles at 7, 10 and 13 years and CMR score at 17 and 24 years, respectively. Variance inflation factor scores for all confounders in the regression models were all ≤5, illustrating that multicollinearity was unlikely. There was a negative association between the DDS at 7 and 10 years and CMR score at 17 years (*β* = –0·64 (95 % CI –1·27, –0·01) *P*_trend_=0·027 for fifth *v*. first DDS quintile at 7 years; *β* = –0·73 (95 % CI –1·35, –0·12) *P*_trend_=0·037 for fifth *v*. first DDS quintile at 10 years).

**Table 4:** Multivariable linear regression analysis models for the associations between the DASH-diet score (DDS) at 7, 10 and 13 years, and CMR score at 17 years in the ALSPAC population for complete case analysis (*n* 1,526)

DASH diet scoring system	CMR score at 17 years									
	Total *n*	Unadjusted			Model 1*			Model 2†		
		*β*	95 % CI	*P*-value	*β*	95 % CI	*P*-value	*β*	95 % CI	*P*-value
**DDS at 7 years**										
Quintile 1	302	Reference			Reference			Reference		
Quintile 2	344	−0·62	−1·17, –0·06	0·029	−0·55	−1·10, 0·001	0·051	−0·56	−1·11, 0·006	0·047
Quintile 3	359	−0·93	−1·48, –0·38	0·001	−0·88	−1·43, 0·33	0·002	−0·79	−1·34, –0·24	0·005
Quintile 4	293	−0·90	−1·47, –0·32	0·002	−0·91	−1·49, –0·34	0·002	−0·75	−1·33, –0·17	0·011
Quintile 5	228	−0·91	−1·53, –0·29	0·004	−0·88	−1·50, –0·26	0·005	−0·64	−1·27, 0·006	0·048
**DDS at 10 years**										
Quintile 1	333	Reference			Reference			Reference		
Quintile 2	319	−0·28	−0·83, 0·27	0·324	−0·18	−0·73, 0·37	0·515	−0·08	−0·63, 0·47	0·780
Quintile 3	340	−0·50	−1·04, 0·05	0·073	−0·41	−0·96, 0·13	0·136	−0·26	−0·80, 0·29	0·353
Quintile 4	305	−0·51	−1·07, 0·05	0·074	−0·41	−0·97, 0·14	0·145	−0·16	−0·73, 0·40	0·576
Quintile 5	229	−1·08	−1·68, –0·47	<0·001	−1·03	−1·63, –0·43	0·001	−0·73	−1·35, –0·12	0·020
**DDS at 13 years**										
Quintile 1	321	Reference			Reference			Reference		
Quintile 2	354	−0·21	−0·75, 0·33	0·456	−0·26	−0·80, 0·28	0·353	−0·16	−0·70, 0·38	0·564
Quintile 3	253	−0·03	−0·62, 0·57	0·927	−0·05	−0·64, 0·54	0·873	0·05	−0·54, 0·64	0·877
Quintile 4	302	−0·36	−0·92, 0·21	0·218	−0·41	−0·97, 0·16	0·156	−0·28	−0·85, 0·29	0·331
Quintile 5	296	−0·51	−1·08, 0·06	0·077	−0·63	−1·20, –0·06	0·030	−0·29	−0·87, 0·30	0·336

CMR, cardiometabolic risk; ALSPAC, Avon Longitudinal Study of Parents and Children; DDS, DASH-diet score; DASH, dietary approaches to stop hypertension; MVPA, moderate-to-vigorous physical activity.

* Adjusted for sex and accuracy of energy reporting at 7, 10 and 13 years.

† As model 1 but also adjusted for grouped age of the mother at delivery, mothers’ highest education level, family highest social class, and MVPA at 11 years.

**Table 5:** Multivariable linear regression analysis models for the associations between the DASH-diet score (DDS) at 7, 10 and 13 years and CMR score at 24 years in the ALSPAC population for complete case analysis (*n* 1,524)

DASH diet scoring system	CMR scoring at 24 years									
	Total *n*	Unadjusted			Model 1*			Model 2†		
		*β*	95 % CI	*P*-value	*β*	95 % CI	*P*-value	*β*	95 % CI	*P*-value
**DDS at 7 years**										
Quintile 1	396	Reference			Reference			Reference		
Quintile 2	233	−0·29	−0·90, 0·33	0·360	−0·25	−0·86, 0·35	0·415	−0·23	−0·83, 0·37	0·454
Quintile 3	379	−0·79	−1·32, –0·26	0·004	−0·80	−1·32, –0·27	0·003	−0·68	−1·20, –0·15	0·012
Quintile 4	222	−0·80	−1·42, –0·18	0·011	−0·82	−1·43, –0·20	0·009	−0·62	−1·23, –0·002	0·049
Quintile 5	294	−1·19	−1·76, 0·62	<0·001	−1·18	−1·75, –0·61	<0·001	−0·92	−1·49, –0·34	0·002
**DDS at 10 years**										
Quintile 1	306	Reference			Reference			Reference		
Quintile 2	334	0·08	−0·50, 0·67	0·785	0·18	−0·40, 0·77	0·537	0·22	−0·36, 0·80	0·452
Quintile 3	351	0·27	−0·31, 0·85	0·367	0·37	−0·20, 0·95	0·204	0·52	−0·06, 1·09	0·078
Quintile 4	232	0·06	−0·58, 0·71	0·855	0·13	−0·51, 0·77	0·686	0·30	−0·34, 0·94	0·363
Quintile 5	301	−0·95	−1·55, –0·34	0·002	−0·91	−1·51, –0·31	0·003	−0·60	−1·20, –0·005	0·052
**DDS at 13 years**										
Quintile 1	314	Reference			Reference			Reference		
Quintile 2	359	0·08	−0·49, 0·65	0·787	0·05	−0·52, 0·62	0·870	0·17	−0·39, 0·74	0·549
Quintile 3	359	−0·09	−0·67, 0·48	0·751	−0·12	−0·69, 0·45	0·670	−0·06	−0·63, 0·51	0·834
Quintile 4	188	−0·56	−1·24, 0·13	0·111	−0·58	−1·26, 0·11	0·099	−0·42	−1·10, 0·26	0·227
Quintile 5	304	−0·77	−1·37, –0·17	0·012	−0·82	−1·41, –0·22	0·008	−0·45	−1·06, 0·15	0·143

CMR, cardiometabolic risk; ALSPAC, Avon Longitudinal Study of Parents and Children; DASH, dietary approaches to stop hypertension; MVPA, moderate-to-vigorous physical activity.

* Adjusted for sex and accuracy of energy reporting at 7, 10 and 13 years.

† As model 1 but also adjusted for grouped age of the mother at delivery, mothers’ highest education level, family highest social class, and MVPA at 11 years.

A similar pattern of results was also observed for the CMR score at 24 years (*β* = –0·92 (95 % CI –1·49, –0·34) *P*_trend_=0·001 for fifth *v*. first DDS quintile at 7 years; *β* = –0·60 (95 % CI –1·20, –0·05) *P*_trend_=0·092 for fifth *v*. first DDS quintile at 10 years). Although the associations observed in the fully adjusted model were weaker than for the partially adjusted models, strong negative associations remained between the DDS at 7 and 10 years and CMR score at 17 and 24 years. No associations were observed between the DDS at 13 years and CMR score at 17 and 24 years in the fully adjusted model. Sensitivity analyses, restricted to the subset of participants with plausible dietary data, showed similar patterns of associations compared with the whole sample in the fully adjusted models (see online supplementary material, Supplementary Tables S5 and S6).

The adjusted associations between the DDS (per five-unit increment) at 7, 10 and 13 years and individual CMR factors (anthropometrics, blood lipids, blood pressure and glucose metabolism) at 17 years and 24 years are also demonstrated in Tables 6 and 7, respectively. A five-unit increment in DDS at 7 and 10 years was negatively associated with anthropometric (BMI and FMI) and glucose metabolism (insulin and HOMA-IR) CMR markers at 17 years. No associations were found between the DDS at 7, 10 and 13 years and blood lipids and blood pressure CMR factors at 17 years. However, a five-unit increment in DDS at 7 and 13 years was associated with a decrease in anthropometric (BMI and FMI) and glucose metabolism measurements (insulin and HOMA-IR) at 24 years. There were also negative associations between the DDS at 7 years and blood pressure (DBP) and blood lipids (HDL-cholesterol) at 24 years.

**Table 6:** Association between the DASH-diet score (DDS) at 7, 10 and 13 years and individual CMR factors at 17 years in the ALSPAC population for complete case analysis (*n* 1,526)

CMR factors at 17 years (*n* 1,526)	DASH diet score (DDS), per 5-unit increment								
	DDS at 7 years			DDS at 10 years			DDS at 13 years		
	*β* *	95 %CI	*P*-value	*β* *	95 %CI	*P*-value	*β* *	95 %CI	*P*-value
***Anthropometric***									
BMI, kg/m^2^	−0·27	–0·46, –0·08	0·006	−0·15	–0·34, 0·03	0·098	−0·14	–0·32, 0·05	0·149
Fat mass index, kg/m^2^ †	−0·28	–0·43, –0·12	<0·001	−0·18	–0·32, –0·03	0·019	−0·16	–0·31, –0·01	0·036
***Blood lipids***									
Total cholesterol, mmol/L	0·00	–0·04, 0·03	0·877	0·01	–0·02, 0·05	0·453	0·01	–0·02, 0·05	0·558
HDL-cholesterol, mmol/l†	0·01	–0·01, 0·02	0·256	0·01	–0·01, 0·02	0·217	0·01	–0·01, 0·02	0·404
LDL-cholesterol, mmol/L†	−0·02	–0·05, 0·02	0·311	0·00	–0·03, 0·03	0·968	−0·00	–0·04, 0·03	0·828
TAG, mmol/l†	0·01	–0·01, 0·03	0·304	0·01	–0·01, 0·03	0·258	0·01	–0·00, 0·03	0·105
***Blood pressure***									
Systolic BP, mmHg	−0·14	–0·65, 0·37	0·591	−0·14	–0·63, 0·35	0·576	−0·09	–0·59, 0·41	0·716
Diastolic BP, mmHg	−0·09	–0·43, 0·26	0·614	−0·24	–0·58, 0·09	0·154	−0·16	–0·50, 0·18	0·361
Mean arterial BP, mmHg†	−0·11	–0·45, 0·24	0·552	−0·21	–0·55, 0·13	0·225	−0·14	–0·48, 0·21	0·437
***Glucose metabolism***									
Insulin, mU/l	−0·34	–0·54, –0·15	0·001	−0·25	–0·44, –0·06	0·010	−0·14	–0·34, 0·05	0·145
Glucose, mmol/l	−0·01	–0·03, 0·01	0·457	0·01	–0·01, 0·03	0·338	0·02	–0·00, 0·03	0·077
HOMA-IR†	−0·08	–0·13, –0·04	0·001	−0·06	–0·10, –0·01	0·014	−0·03	–0·07, 0·02	0·216

CMR, cardiometabolic risk; ALSPAC, Avon Longitudinal Study of Parents and Children; DASH, dietary approaches to stop hypertension; BP, blood pressure; HOMA-IR, homoeostatic model assessment of insulin resistance; MVPA, moderate-to-vigorous physical activity.

* Beta coefficients (95 % CI) derived from multivariable linear regression models adjusted for sex, plausibility of dietary energy intake at 7, 10 or 13 years, maternal age at delivery, maternal highest education level, family highest social class and MVPA at 11 years.

† Cardiometabolic variables included in the cardiometabolic risk score.

**Table 7:** Association between the DASH-diet score (DDS) at 7, 10 and 13 years and individual CMR factors at 24 years in the ALSPAC population for complete case analysis (*n* 1,524)

CMR factors at 24 years (*n* 1,524)	DASH diet score (DDS), per 5-unit increment								
	DDS at 7 years			DDS at 10 years			DDS at 13 years		
	*β* *	95 %CI	*P*-value	*β* *	95 %CI	*P*-value	*β* *	95 %CI	*P*-value
***Anthropometric***									
BMI, kg/m^2^	−0·32	–0·55, –0·08	0·008	−0·08	–0·31, 0·14	0·469	−0·26	–0·48, –0·03	0·028
Fat mass index, kg/m^2^ †	−0·29	–0·46, –0·12	0·001	−0·11	–0·27, 0·06	0·203	−0·25	–0·42, 0·09	0·003
***Blood lipids***									
Total cholesterol, mmol/L	−0·00	–0·04, 0·04	0·976	−0·00	–0·04, 0·04	0·973	−0·00	–0·04, 0·04	0·920
HDL-cholesterol, mmol/l†	0·03	0·00, 0·05	0·018	0·01	–0·01, 0·03	0·355	0·01	0·01, 0·03	0·488
LDL-cholesterol, mmol/l†	−0·03	–0·07, 0·01	0·168	−0·01	–0·05, 0·03	0·560	−0·01	–0·05, 0·03	0·608
TAG, mmol/l†	0·00	–0·02, 0·03	0·673	0·00	–0·02, 0·02	0·816	0·00	–0·02, 0·02	0·873
***Blood pressure***									
Systolic BP, mmHg	−0·43	–0·96, 0·10	0·111	−0·08	–0·59, 0·43	0·748	−0·55	–1·06, –0·03	0·037
Diastolic BP, mmHg	−0·52	–0·92, –0·11	0·012	−0·18	–0·57, 0·21	0·354	−0·28	–0·68, –0·11	0·155
Mean arterial BP, mmHg†	−0·49	–0·89, –0·08	0·018	−0·15	–0·54, 0·24	0·449	−0·37	–0·76, –0·02	0·064
***Glucose metabolism***									
Insulin, mU/l	−0·47	–0·76, –0·18	0·002	−0·24	–0·52, 0·04	0·098	−0·30	–0·58, 0·02	0·038
Glucose, mmol/l	0·00	–0·02, 0·03	0·752	0·00	–0·02, 0·02	0·948	−0·01	–0·03, 0·01	0·460
HOMA-IR†	−0·12	–0·19, –0·04	0·002	−0·06	–0·13, 0·01	0·117	−0·08	–0·15, –0·01	0·029

CMR, cardiometabolic risk; ALSPAC, Avon Longitudinal Study of Parents and Children; DASH, dietary approaches to stop hypertension; BP, blood pressure; HOMA-IR, homoeostatic model assessment of insulin resistance; MVPA, moderate-to-vigorous physical activity.

* Beta coefficients (95 % CI) derived from multivariable linear regression models adjusted for sex, plausibility of dietary energy intake at 7, 10 or 13 years, maternal age at delivery, maternal highest education level, family highest social class and MVPA at 11 years.

† Cardiometabolic variables included in the cardiometabolic risk score.

## Discussion

To the best of our knowledge, this is the first prospective study to assess whether a DASH-style dietary pattern in childhood is associated with overall cardiometabolic health in adolescents and young adults. Our findings showed that higher DDS at 7 and 10 years were associated with lower CMR scores at 17 and 24 years. These associations between the DASH diet and CMR scores were largely driven by decreased adiposity and insulin resistance at 17 years and by blood pressure and HDL-cholesterol reductions at 24 years. These results suggest that greater accordance with a DASH-style diet during childhood could lead to better cardiometabolic health outcomes in adolescence/early adulthood.

Our results are in contrast to the UK population-based study using National Diet and Nutrition Survey data, which did not observe an association between the DASH diet and a metabolic risk score in the 11–18- or 19–35-year-old age groups, although they did find a strong association in the 36–60-year-old age group (*β* = –0·27 (95 % CI –0·39, –0·16))^(15)^. These contrasting findings may be partly explained by differences in study methodology between their study and ours, such as their cross-sectional design, smaller sample size within the age-specific analyses and several differences in the metabolic markers included in their risk score (waist circumference and glucose levels compared with FMI and HOMA-IR in our study). It should be noted, however, that we did not find an association between the DDS at 13 years and CMR at 17 or 24 years. One explanation could be that higher adherence to DASH-style diets in earlier childhood (7–10 years) compared with teenage years (13 years) had a greater influence on cardiometabolic risk, indicating a potentially more sensitive period. It might also reflect increased dietary misreporting in adolescents, particularly of socially undesirable foods^(44)^ or greater difficulty capturing habitual diets due to more changeable diets during this period^(45,46)^.

In terms of how the DASH-style diet was related to individual CMR factors, children with higher DDS at most ages had a lower BMI and/or FMI at 17 and 24 years. These strong associations with adiposity are in accordance with overall findings from a systematic review on the DASH diet and adiposity in adolescence^(25)^. For example, a US cohort study included in this review found that young girls (*n* 2,327) who adhered more closely to the DASH diet had a 1·9 kg/m^2^ lower adjusted mean BMI than girls with lower adherence^(27)^. Our study also found that the DASH-style diet at 7 years was related to lower BP at 24 years. This association was not evident at 17 years though: this might be due to the smaller variation in BP values between participants at 17 years than at 24 years (data not shown) that could have reduced the power to detect individual CMR marker associations at the earlier age. Findings from this US cohort also showed that 9–10-year-old girls (*n* 2,185) who adhered more closely to a DASH-style diet had a 36 % (95 % CI: 3 %, 57 %) reduced risk of higher BP in early adulthood (18–20 years)^(26)^. The reductions in BP and BMI were mainly due to the high consumption of fruit and vegetables, low-fat dairy products and whole grains. The cross-sectional UK study using National Diet and Nutrition Survey data also found a fruit and vegetable biomarker score was related to overall metabolic risk^(15)^. In our study the DASH-style diet was also associated with a decrease in fasting insulin levels, which is supported by findings from controlled trials in adults^(16)^.

Several characteristics of the DASH-style dietary pattern may explain our findings. A high consumption of fruits and vegetables, wholegrains, nuts and legumes could increase satiety due to their high fibre and water content^(47)^. This could reduce EI, leading to lower adiposity due to the lower energy density of this dietary pattern. Similarly, dairy-derived proteins can also induce satiety and reduce the glycaemic response to different carbohydrates through the stimulation of different appetite hormones^(48)^. Fruits and vegetables and low-fat dairy products can also reduce BP by promoting vasodilation and better regulation of the vascular system^(26,49)^. These foods are rich sources of antioxidants (fruit and vegetables) and nutrients such as Ca, potassium and Mg, which may relax the vascular smooth muscle, promote natriuresis and activate the renin–angiotensin–aldosterone and adrenergic systems^(16,50)^.

The longitudinal design covering up to 17 years of follow-up, relatively large sample size and adjustment for relevant confounders are key strengths of this study. Another advantage is that the DASH diet was assessed at three different time points throughout childhood (7, 10 and 13 years) and therefore covered the transition period into adolescence, a period that can result in shifts in dietary habits due to increased autonomy in food choices outside the home^(45,46)^. There might also be critical periods during childhood when dietary patterns are more influential on future cardiometabolic health. Using CMR scores was also beneficial because it provides a representation of participants’ overall cardiometabolic health and can be used as an intermediate pre-clinical measure of risk in younger populations before overt CMD are present. Furthermore, CMR scores are found to be better predictors of CVD in children compared to individual dichotomous measures, illustrating an important outcome for prevention purposes in children^(29)^.

A further strength is the use of accelerometers to directly measure physical activity, as this has been shown to be a more valid, objective and reliable method than self-reported physical activity^(51)^. Research into dietary patterns such as the DASH-style diet is also advantageous as it captures the combined effects of food groups/nutrients whose separate effects on health may be undetectable. In addition, findings from research on dietary patterns may be more practical to inform dietary recommendations than research into single foods or nutrients. In fact, the DASH dietary pattern aligns closely with many of the dietary recommendations within the UK’s Eatwell Guide^(52)^, as they both encourage consumption of plant-based foods, such as fruit and vegetables, and pulses, wholegrain foods and low-fat dairy products, and limiting foods high in salt/sugar and red and processed meat.

A limitation of this study is loss to follow-up bias, which meant that certain characteristics of the final study sample differed from the original ALSPAC population at birth. For example, the study population had a higher household social class, higher maternal educational levels and better cardiometabolic health at 17 and 24 years than those who were excluded from the study due to incomplete data. Previous research in ALSPAC^(53)^ found an association between socio-economic status and dietary patterns in the ALSPAC population, meaning that children with less healthy dietary patterns were probably under-represented in our cohort. Thus, the generalisability of the strength of associations observed in our study to the overall UK population should be taken with caution. Another potential limitation of this study is the use of baseline maternal and paternal occupation to classify social class, included as a confounding variable, since this may have changed over the duration of the study. The methodology behind the DDS should also be considered, since the different components of the DDS are given similar weighting within the score, assuming they have an equal relationship with cardiometabolic health. In addition, the DASH diet was defined based on the distribution of dietary intakes in the study populations and not on specific recommended amounts. Therefore, participants in the highest quintile of the DDS may have different dietary intakes to participants in the highest quintile from studies in different countries. However, this would not affect the internal validity of the observed associations. Similarly, the CMR score is population specific as it is based on the distribution of cardiometabolic risk markers within our study population. Although we adjusted for a range of relevant confounders, including plausibility of dietary reporting, it is possible there was residual confounding due to measurement error in these data, or other confounding factors not included.

In conclusion, this study demonstrated that greater adherence to a DASH-style dietary pattern during childhood (7–10 years) was associated with better cardiometabolic health in adolescence and early adulthood in the ALSPAC cohort. Previous research has shown that these CMR factors are predictive of developing CMD^(30)^. This highlights that DASH-style healthy dietary patterns, commencing from childhood, are important for preventing the early onset of different CMR factors, and this may potentially reduce the risk of developing CMD later in life. Advocating the key attributes of the DASH diet to children and adolescents with emerging CMR factors may also be useful in a clinical setting. Similar studies are still needed to validate our findings, particularly from paediatric populations in different countries.

## Supporting information

Loizou et al. supplementary material Loizou et al. supplementary material

## Data Availability

The data the study is based upon are available by application on the ALSPAC website: http://www.bristol.ac.uk/alspac/researchers/access/.

## Supplementary material

For supplementary material accompanying this paper visit https://doi.org/10.1017/S136898002400048X.
