# Supplementary material for: The dietary approaches to stop hypertension (DASH) dietary pattern in childhood in relation to cardiometabolic risk in adolescence and early adulthood in the ALSPAC birth cohort
Source: Public Health Nutr. 2024 Mar 21;27(1):e86. doi: 10.1017/S136898002400048X (PMC10966836; doi:10.1017/S136898002400048X)
Supplement: Loizou et al. supplementary material [file S136898002400048Xsup001.docx]

# Supplementary Material: Public Health Nutrition

**The DASH-style dietary pattern in childhood in relation to cardiometabolic risk in adolescence and early adulthood in the ALSPAC birth cohort.**

## Supplementary Tables

**Supplementary Table S1:** Details of the foods, nutrients and beverages included within the eight dietary components of the DASH diet score (DDS) in the present study.

| **DDS dietary components** | **Foods or beverages included** | **Notes** |
| --- | --- | --- |
| Fruits | All fruits (e.g., citrus fruit, apples, pears, and bananas) fresh and packaged juices, dried fruit, and stewed fruit. | Fruit canned in juice and in syrup were also included. |
| Vegetables | All vegetables (e.g., raw carrots, cooked carrots, green leafy vegetables, green beans, fried vegetables, raw tomatoes, other cooked vegetables and salad, and raw vegetables). | Potatoes and peas were excluded, and mixed vegetables were included. |
| Nuts and legumes | Nuts, seeds, legumes, soya products, soya milk (8% net weight), peanut butter (smooth), peanut butter (wholegrain), peanut butter (smooth and wholegrain), and baked beans (50% net weight). | Peanut butters smooth and wholegrain were separated from broader food groups. |
| Whole grains | Brown and granary bread, brown rice (boiled), popcorn (plain), spaghetti (wholemeal). | Wholegrain breakfast cereals were also included. Brown rice, popcorn, and spaghetti were separated from broader food groups. |
| Low-fat dairy | Low-fat milk, yogurt and fromage frais (low-fat), cheese and cheese dishes (low-fat), polyunsaturated margarine (low-fat), and polyunsaturated margarine (low-fat). | Yogurt and fromage frais (low-fat), and cheese and cheese dishes (low-fat) were created after separating items from broader groups. |
| Red and processed meat | Processed meat, ham, bacon, sausages, liver and dishes, meat and poultry, meat mixed dishes, meat pies, pastries, burgers, and kebabs | Meat and poultry, meat mixed dishes, meat pies, pastries, burgers, and kebabs were created after mixed food disaggregation. |
| Non-milk extrinsic sugars | Table sugar, glucose, fructose, honey, syrups, added sugar to foods, and beverages. | This dietary component was included by Jones et al.,^(^[^12^](#_ENREF_1_12)^)^ from the original DDS to better represent the DASH diet. |
| Sodium | Sodium/salt | Sodium was estimated based on the amount consumed in foods or drinks. |

Abbreviations: DDS, DASH diet score; DASH, dietary approaches to stop hypertension.

**Supplementary Table S2:** Comparison of baseline characteristics in ALSPAC participants included and excluded from the current study. Participants included had complete dietary data at all ages (7, 10 and 13 years) and outcome data at 17 years and/or 24 years, and participants excluded had missing data on these variables.

| **Characteristics of the ALSPAC population** | **Total n** | **Participants included (n = 2,738)** | | **Participants excluded (n = 11,908)** | | **P-value^*^** |
| --- | --- | --- | --- | --- | --- | --- |
|  |  | **n** | **% or median (IQR)** | **n** | **% or median (IQR)** |  |
| Sex, male | 7508 | 1281 | 46.8 | 6227 | 52.3 |  |
| Sex, female | 7138 | 1457 | 53.2 | 5681 | 47.7 | <0.001 |
| Energy intake (kJ/day) |  |  |  |  |  |  |
| 7 years | 7259 | 2738 | 7164 (6355-8006) | 4521 | 7038 (6218-7939) | <0.001 |
| 10 years | 7444 | 2738 | 7778 (6827-8830) | 4706 | 7622 (6674-8733) | <0.001 |
| 13 years | 6089 | 2738 | 8061 (6805-9548) | 3351 | 7966 (6612-9502) | 0.030 |
| Child BMI at 10 years (kg/m^2^) | 7448 | 2719 | 17.4 (15.93-19.52) | 4729 | 17.5 (16.04-20.01) | 0.003 |
| Grouped age of mother at delivery (years) | 13,930 | 2631 | 30.0 (27.0-33.0) | 11,299 | 27.0 (24.0-31.0) | <0.001 |
| Mothers BMI (kg/m^2^) | 11,495 | 2449 | 22.0 (20.5-24.0) | 9046 | 22.2 (20.5-24.5) | 0.004 |
| Maternal highest education^†^ |  |  |  |  |  |  |
| A-level or Degree | 8001 | 1243 | 52.1 | 3021 | 30.9 |  |
| CSE, Vocational or O-level | 4372 | 1351 | 47.9 | 6758 | 69.1 | <0.001 |
| Highest household social class^‡^ |  |  |  |  |  |  |
| Grade I and II | 2975 | 877 | 34.7 | 2098 | 23.6 |  |
| Grade III, IV and V | 8456 | 1654 | 65.4 | 6802 | 76.4 | <0.001 |
| Moderate-to-vigorous PA at 11 years |  |  |  |  |  |  |
| <20 mins | 3022 | 1194 | 51.2 | 1828 | 50.8 |  |
| ≥20 mins | 2912 | 1140 | 48.8 | 1772 | 49.2 | 0.775 |
| Accuracy of energy reporting at 7 years^§^ |  |  |  |  |  |  |
| Misreporting | 1820 | 650 | 23.8 | 1170 | 26.2 |  |
| Valid | 5372 | 2078 | 76.2 | 3294 | 73.8 | 0.024 |

Abbreviations: DDS, DASH diet score; DASH, dietary approaches to stop hypertension; IQR, interquartile range; BMI, body mass index; CSE, certificate of secondary education; PA, physical activity.

*p values were calculated using the Kruskal-Wallis test and the Pearson correlation coefficient test.

^†‡§^The following categorical variables including maternal highest education (A-level or degree and CSE, vocational or O-level), highest household social class (grade I or II, and grade III, IV or V) and accuracy of energy reporting (misreporting and valid) were recategorized for the purpose of the above table.

| **Supplementary Table S3:** Comparison of cardiometabolic risk (CMR) factors at 17 and 24 years in ALSPAC participants included and excluded from the current study. Participants included had complete dietary data at all ages (7, 10 and 13 years) and outcome data at 17 years and/or 24 years, and participants excluded had missing data on these variables. |
| --- |

| **Characteristics of the ALSPAC population** | **Total n** | **Participants included (n=2738)** | | **Participants excluded (n=11,908)** | | **P-value^*^** |
| --- | --- | --- | --- | --- | --- | --- |
|  |  | **n** | **Median (IQR)** | **n** | **Median (IQR)** |  |
| **CMR factors at 17 years** | | | | | | |
| FMI (kg/m^2^) | 4821 | 2444 | 5.54 (3.30-7.84) | 2,377 | 6.08 (3.84-8.68) | <0.001 |
| HDL-c (mmol/L) | 3281 | 2076 | 1.24 (1.06-1.45) | 1,205 | 1.22 (1.04-1.43) | 0.020 |
| LDL-c (mmol/L) | 3281 | 2076 | 2.04 (1.66-2.47) | 1,205 | 2.04 (1.69-2.47) | 0.524 |
| TG (mmol/L) | 3281 | 2076 | 0.74 (0.60-0.96) | 1,205 | 0.78 (0.61-1.01) | 0.002 |
| MAP (mmHg) | 4670 | 2426 | 81.00 (77.17-85.67) | 2,244 | 81.50 (77.67-87.00) | 0.002 |
| HOMA-IR | 3211 | 2055 | 1.46 (1.06-2.04) | 1,156 | 1.59 (1.12-2.43) | <0.001 |
| **CMR factors at 24 years** | | | | | | |
| FMI (kg/m^2^) | 3766 | 2100 | 6.87 (5.28-9.35) | 1,666 | 7.50 (5.67-10.11) | <0.001 |
| HDL-c (mmol/L) | 3178 | 2019 | 1.52 (1.26-1.79) | 1,159 | 1.50 (1.24-1.79) | 0.562 |
| LDL-c (mmol/L) | 3176 | 2019 | 2.37 (1.91-2.89) | 1,157 | 2.35 (1.92-2.91) | 0.810 |
| TG (mmol/L) | 3177 | 2019 | 0.83 (0.65-1.13) | 1,158 | 0.84 (0.66-1.19) | 0.077 |
| MAP (mmHg) ^†^ | 3908 | 2141 | 82.67 (77.78-88.33) | 1,767 | 82.67 (77.50-88.44) | 0.805 |
| HOMA-IR^‡^ | 3178 | 2019 | 1.72 (1.20-2.51) | 1,159 | 1.81 (1.23-2.77) | 0.015 |

Abbreviations: DDS, DASH diet score; DASH, dietary approaches to stop hypertension; CMR, cardiometabolic risk; FMI, fat mass index; HDL-c, high-density lipoprotein cholesterol; LDL-c, low-density lipoprotein cholesterol; TG, triacylglycerol; MAP, mean arterial blood pressure; HOMA-IR, homeostatic model assessment of insulin resistance.
^*^p values were calculated using the Kruskal-Wallis test and the Pearson correlation coefficient test.
^†^MAP = (SBP+(2×DBP))/3.
^‡^HOMA-IR = fasting plasma glucose (mg/dl) ×fasting plasma insulin (mU/L))/405.

**Supplementary Table S4:** Representation of the R-squared statistic for the crude analysis and the models 1 and 2 between the DASH-diet score (DDS) at 7, 10 and 13 years and cardiometabolic risk (CMR) score at 17 and 24 years.

|  | **Unadjusted** | **Model 1^*^** | **Model 2**^†^ |
| --- | --- | --- | --- |
| **CMR scoring at 17 years** |  |  |  |
| DDS at 7 years | 0.008 | 0.016 | 0.042 |
| DDS at 10 years | 0.007 | 0.020 | 0.042 |
| DDS at 13 years | 0.003 | 0.021 | 0.049 |
| **CMR scoring at 24 years** |  |  |  |
| DDS at 7 years | 0.013 | 0.028 | 0.055 |
| DDS at 10 years | 0.013 | 0.026 | 0.056 |
| DDS at 13 years | 0.008 | 0.021 | 0.052 |

Abbreviations: DDS, DASH diet score; DASH, dietary approaches to stop hypertension; CMR, cardiometabolic risk.
^*^Model 1: Adjusted for sex and accuracy of energy reporting at 7, 10 and 13 years.
^†^Model 2: As model 1 but also adjusted maternal age at delivery, maternal highest education level, family highest social class and MVPA at 11 years.

**Supplementary Table S5.** Multivariable linear regression analysis models for the associations between the DASH-diet score (DDS) at 7, 10 and 13 years, and cardiometabolic risk (CMR) score at 17 years in the whole cohort and restricted to plausible dietary reporters from the ALSPAC population.

| **DASH diet scoring system** | **CMR score at 17 years** | | | | | | | | |
| --- | --- | --- | --- | --- | --- | --- | --- | --- | --- |
|  | **All cohort** | | | |  | **Plausible dietary reporters** | | | |
|  | **Total n** | **Beta*** | **95% CI** | **P-value** |  | **Total n** | **Beta*** | **95% CI** | **P-value** |
| **DDS at 7 years** | | | | |  |  |  |  |  |
| Quintile 1 | 302 | Reference | | |  | 228 | Reference |  |  |
| Quintile 2 | 344 | -0.56 | -1.11, 0.006 | 0.047 |  | 245 | -0.85 | -1.49, -0.20 | 0.010 |
| Quintile 3 | 359 | -0.79 | -1.34, -0.24 | 0.005 |  | 285 | -1.07 | -1.70, -0.45 | 0.001 |
| Quintile 4 | 293 | -0.75 | -1.33, -0.17 | 0.011 |  | 228 | -0.91 | -1.58, -0.25 | 0.007 |
| Quintile 5 | 228 | -0.64 | -1.27, 0.006 | 0.048 |  | 176 | -0.55 | -1.27, 0.17 | 0.136 |
|  |  |  |  |  |  |  |  |  |  |
| **DDS at 10 years** | | | | |  |  |  |  |  |
| Quintile 1 | 333 | Reference | | |  | 201 | Reference | | |
| Quintile 2 | 319 | -0.08 | -0.63, 0.47 | 0.780 |  | 228 | -0.41 | -1.04, 0.22 | 0.207 |
| Quintile 3 | 340 | -0.26 | -0.80, 0.29 | 0.353 |  | 245 | -0.63 | -1.25, -0.01 | 0.047 |
| Quintile 4 | 305 | -0.16 | -0.73, 0.40 | 0.576 |  | 219 | -0.69 | -1.34, -0.04 | 0.039 |
| Quintile 5 | 229 | -0.73 | -1.35, -0.12 | 0.020 |  | 150 | -0.86 | -1.58, -1.15 | 0.018 |
|  |  |  |  |  |  |  |  |  |  |
| **DDS at 13 years** | | | | |  |  |  |  |  |
| Quintile 1 | 321 | Reference | | |  | 129 | Reference | | |
| Quintile 2 | 354 | -0.16 | -0.70, 0.38 | 0.564 |  | 136 | -0.39 | -1.13, 0.35 | 0.305 |
| Quintile 3 | 253 | 0.05 | -0.54, 0.64 | 0.877 |  | 104 | -0.01 | -0.80, 0.78 | 0.986 |
| Quintile 4 | 302 | -0.28 | -0.85, 0.29 | 0.331 |  | 125 | -0.22 | -0.99, 0.55 | 0.577 |
| Quintile 5 | 296 | -0.29 | -0.87, 0.30 | 0.336 |  | 106 | 0.73 | -1.54, 0.87 | 0.080 |
| Abbreviations: DDS, DASH-diet score; DASH, dietary approaches to stop hypertension; CMR, cardiometabolic risk.  *Adjusted for sex, accuracy of energy reporting at 7, 10 and 13 years, grouped age of the mother at delivery, mothers’ highest education level, family highest social class, and MVPA at 11 years. | | | | | | | | | |

**Supplementary Table S6.** Multivariable linear regression analysis models for the associations between the DASH-diet score (DDS) at 7, 10 and 13 years, and cardiometabolic risk (CMR) score at 24 years in the whole cohort and restricted to plausible dietary reporters from the ALSPAC population.

| **DASH diet scoring system** | **CMR score at 24 years** | | | | | | | | |
| --- | --- | --- | --- | --- | --- | --- | --- | --- | --- |
|  | **All cohort** | | | |  | **Plausible dietary reporters** | | | |
|  | **Total n** | **Beta*** | **95% CI** | **P-value** |  | **Total n** | **Beta*** | **95% CI** | **P-value** |
| **DDS at 7 years** | | | | |  |  |  |  |  |
| Quintile 1 | 396 | Reference |  |  |  | 302 | Reference |  |  |
| Quintile 2 | 233 | -0.23 | -0.83, 0.37 | 0.454 |  | 171 | -0.15 | -0.84, 0.53 | 0.664 |
| Quintile 3 | 379 | -0.68 | -1.20, -0.15 | 0.012 |  | 304 | -0.82 | -1.40, -0.23 | 0.006 |
| Quintile 4 | 222 | -0.62 | -1.23, -0.002 | 0.049 |  | 169 | -0.86 | -1.55, -0.17 | 0.015 |
| Quintile 5 | 294 | -0.92 | -1.49, -0.34 | 0.002 |  | 233 | -1.08 | -1.72, -0.44 | 0.001 |
|  |  |  |  |  |  |  |  |  |  |
| **DDS at 10 years** | | | | |  |  |  |  |  |
| Quintile 1 | 306 | Reference |  |  |  | 180 | Reference |  |  |
| Quintile 2 | 334 | 0.22 | -0.36, 0.80 | 0.452 |  | 227 | -0.05 | -0.75, 0.66 | 0.891 |
| Quintile 3 | 351 | 0.52 | -0.06, 1.09 | 0.078 |  | 248 | 0.25 | -0.45, 0.95 | 0.482 |
| Quintile 4 | 232 | 0.30 | -0.34, 0.94 | 0.363 |  | 151 | -0.17 | -0.96, 0.61 | 0.665 |
| Quintile 5 | 301 | -0.60 | -1.20, -0.005 | 0.052 |  | 191 | -1.02 | -1.77, -0.27 | 0.007 |
|  |  |  |  |  |  |  |  |  |  |
| **DDS at 13 years** | | | | |  |  |  |  |  |
| Quintile 1 | 314 | Reference |  |  |  | 128 | Reference |  |  |
| Quintile 2 | 359 | 0.17 | -0.39, 0.74 | 0.549 |  | 146 | 0.34 | -0.50, 1.17 | 0.427 |
| Quintile 3 | 359 | -0.06 | -0.63, 0.51 | 0.834 |  | 145 | -0.11 | -0.95, 0.73 | 0.799 |
| Quintile 4 | 188 | -0.42 | -1.10, 0.26 | 0.227 |  | 81 | -0.23 | -1.22, 0.76 | 0.651 |
| Quintile 5 | 304 | -0.45 | -1.06, 0.15 | 0.143 |  | 120 | -0.57 | -1.49, 0.34 | 0.218 |
| Abbreviations: DDS, DASH-diet score; DASH, dietary approaches to stop hypertension; CMR, cardiometabolic risk.  *Adjusted for sex, accuracy of energy reporting at 7, 10 and 13 years, grouped age of the mother at delivery, mothers’ highest education level, family highest social class, and MVPA at 11 years. | | | | | | | | | |
